# Baseline cognition is the best predictor of 4-year cognitive change in cognitively intact older adults

**DOI:** 10.1186/s13195-021-00798-4

**Published:** 2021-04-07

**Authors:** Jolien M. Schaeverbeke, Silvy Gabel, Karen Meersmans, Emma S. Luckett, Steffi De Meyer, Katarzyna Adamczuk, Natalie Nelissen, Valerie Goovaerts, Ahmed Radwan, Stefan Sunaert, Patrick Dupont, Koen Van Laere, Rik Vandenberghe

**Affiliations:** 1 Laboratory for Cognitive Neurology, Department of Neurosciences, Leuven Brain Institute, KU Leuven, Leuven, Belgium; 2 Neurology Department, University Hospitals Leuven, Herestraat 49, Leuven, 3000 Belgium; 3 Laboratory of Molecular Neurobiomarker Research, KU Leuven, Leuven, Belgium; 4 Translational MRI, Department of Imaging and Pathology, KU Leuven, Leuven, Belgium; 5 Nuclear Medicine and Molecular Imaging, Department of Imaging and Pathology, KU Leuven and Division of Nuclear Medicine, University Hospitals Leuven, Leuven, Belgium

**Keywords:** Alzheimer’s disease, Cognitive decline, Mediation, Preclinical, Ageing

## Abstract

**Background:**

We examined in cognitively intact older adults the relative weight of cognitive, genetic, structural and amyloid brain imaging variables for predicting cognitive change over a 4-year time course.

**Methods:**

One hundred-eighty community-recruited cognitively intact older adults (mean age 68 years, range 52–80 years, 81 women) belonging to the Flemish Prevent Alzheimer’s Disease Cohort KU Leuven (F-PACK) longitudinal observational cohort underwent a baseline evaluation consisting of detailed cognitive assessment, structural MRI and ^18^F-flutemetamol PET. At inclusion, subjects were stratified based on Apolipoprotein E (*APOE*) *ε*4 and Brain-Derived Neurotrophic Factor (*BDNF*) val66met polymorphism according to a factorial design. At inclusion, 15% were amyloid-PET positive (Centiloid >23.4). All subjects underwent 2-yearly follow-up of cognitive performance for a 4-year time period. Baseline cognitive scores were analysed using factor analysis. The slope of cognitive change over time was modelled using latent growth curve analysis. Using correlation analysis, hierarchical regression and mediation analysis, we examined the effect of demographic (age, sex, education) and genetic variables, baseline cognition, MRI volumetric (both voxelwise and region-based) as well as amyloid imaging measures on the longitudinal slope of cognitive change.

**Results:**

A base model of age and sex explained 18.5% of variance in episodic memory decline. This increased to 41.6% by adding baseline episodic memory scores. Adding amyloid load or volumetric measures explained only a negligible additional amount of variance (increase to 42.2%). A mediation analysis indicated that the effect of age on episodic memory scores was partly direct and partly mediated via hippocampal volume. Amyloid load did not play a significant role as mediator between age, hippocampal volume and episodic memory decline.

**Conclusion:**

In cognitively intact older adults, the strongest baseline predictor of subsequent episodic memory decline was the baseline episodic memory score. When this score was included, only very limited explanatory power was added by brain volume or amyloid load measures. The data warn against classifications that are purely biomarker-based and highlight the value of baseline cognitive performance levels in predictive models.

## Introduction

There has been a longstanding interest in the brain mechanisms underlying cognitive ageing. One of the key characteristics of cognitive ageing [1] is decline in episodic memory performance [2]. This decline starts around the third decade [3]. The rate of decline is relatively constant within an individual under normal circumstances [3–5] but highly variable between individuals [1]. Fluid intelligence, processing speed and working memory show a similar age-related decline [3].

With the advent of Alzheimer’s disease (AD) in vivo imaging biomarkers, it has become possible to test to which degree ‘asymptomatic’ [6] or ‘preclinical’ AD [7] contributes to the variance in episodic memory decline in older adults who fall within published neuropsychological test norms. Possibly, age-related changes in cognition in cognitively intact individuals could be driven by subclinical AD processes, for which age is the most important risk factor [8]. Increased amyloid load in cognitively intact healthy older adults is associated with slightly lower baseline cognitive scores and a higher risk of cognitive decline over the subsequent years [9–14].

In this longitudinal study, we examined which variables predict the subsequent 4-year time course of cognitive functioning in older adults. Per protocol, baseline cognitive scores had to be within the normal range for inclusion. At inclusion, cases were stratified for *APOE* *ε*4 and *BDNF* codon 66 *met* carrier status according to a factorial design. The genetic stratification was meant to enrich the cohort for risk of developing AD as *APOE* *ε*4 is a highly prevalent and potent genetic risk factor for AD [15] and a predictor of the rate of cognitive decline in patients with AD [16]. Moreover, recent work has indicated a higher rate of cognitive decline in *BDNF* val66met carriers who are amyloid-positive [17].

Like most brain structures, apart from brainstem, hippocampal volume decreases with age [18–21]. It has been hypothesized that reduced episodic memory with older age is mediated by hippocampal volume loss [18, 22], and that the hippocampal volume loss is partly a consequence of increased brain amyloidosis [23]. We tested these hypotheses by means of mediation analysis.

## Materials and methods

### Participants

The Flemish Prevent AD Cohort KU Leuven (F-PACK) is a community-recruited longitudinal cohort of older adults who had to be cognitively intact at inclusion per protocol. The uniqueness derives from the genetic stratification at study inclusion according to a factorial design, with two factors: *APOE* *ε*4 carrier status (two levels: carrier versus noncarrier) and *BDNF* val66met carrier status (two levels: carrier versus noncarrier). Stratification was done so that each cell of the factorial design contained an equal number of participants per 5-year age bin and that cells were also matched for age, educational level and sex. The data from the full cohort have been shared with the EMIF1000 cohort study [24]. The current publication is the first stand-alone detailed description of the full F-PACK cohort. Substudies have been published in subgroups of the F-PACK cohort [25–28].

The a priori target sample size was 180 cognitively intact older controls (mean age = 68.6 years, SD = 6.4 years, Table 1), who were recruited through advertisement in local newspapers and through websites for seniors, asking for healthy volunteers between 50 and 80 years of age for participation in a scientific study at the University Hospital Leuven, Belgium, involving brain imaging (*sic*). Inclusion criteria were age 50–80 years, Mini Mental State Examination (MMSE) [29] ≥27, Clinical Dementia Rating Scale (CDR) = 0 and test scores on neuropsychological assessment within published norms. Among the exclusion criteria were a neurological or psychiatric history and focal brain lesions on structural magnetic resonance image (MRI) other than vascular white matter lesions, e.g. lesions as a result of large-vessel stroke, arachnoid cyst or surgical interventions. Recruitment took place between March 2009 and December 2015.

**Table 1:** Baseline characteristics of the F-PACK cohort

	Total	%	
Sex (male/female)	99/81	55	
CDR 0	180	100	
*APOE* *ε*4 heterozygotes	81	45	
*APOE* *ε*4 homozygotes	6	3.33	
*APOE* *ε*2*ε*3 carriers	16	8.9	
*BDNF* codon 66 *met* carriers	87	48.3	
	Mean	S.D.	Range
Age (years)	68.6	6.4	52.4–80.9
Education (years)	14.2	3.4	8.0–23.5
MMSE (/30)	29.1	0.9	27.0–30.0
AVLT TL (/75)	47.3	9.1	26.0–69.0
AVLT %DR	84.5	17.9	30.0–200.0
BSRT TR (/12)	8.1	1.3	4.8–10.8
BSRT DR (/12)	8.0	2.7	1.0–12.0
BNT (/60)	54.6	4.4	38.0–60.0
AVF (# words)	22.5	5.6	9.0–42.0
LVF (# words)	36.3	11.8	9.0–71.0
PALPA49 (/30)	27.2	1.7	20.0–30.0
RPM (/60)	41.7	8.8	15.0–58.0
TMT B/A	2.5	0.9	0.0–6.7

*APOE*, apolipoprotein E; *BDNF*, Brain-Derived Neurotrophic Factor; *CDR,* Clinical Dementia Rating Scale total score; *MMSE*, Mini Mental State Examination; *AVLT*, Rey Auditory Verbal Learning Test: *total learning* (AVLT TL) and *percentage delayed recall* (AVLT %DR); *BSRT*, Buschke Selective Reminding Test: *total retention* (BSRT TR) and the *delayed recall* (BSRT DR); *BNT*, Boston Naming Test; *AVF*, Animal Verbal Fluency; *LVF*, Letter Verbal Fluency Test; *PALPA*, Psycholinguistic Assessment of Language Processing in Aphasia (PALPA) subtest 49; *RPM*, Raven’s Progressive Matrices; *TMT*, Trail Making Test part B divided by part A

Following baseline, participants are followed 2-yearly with a neuropsychological evaluation consisting of cognitive testing on episodic memory, language, executive functioning and attention domains for a total period of 10 years. At the time of writing of the manuscript, all subjects had completed the 4-year timepoint.

### Cognition

All participants underwent detailed cognitive examination of verbal episodic memory function by means of Rey’s Auditory Verbal Learning Test (AVLT) [30] and the 12-item Buschke Selective Reminding test (BSRT) [31]. From the AVLT two performance parameters were derived: total learning (AVLT TL), i.e. the sum of items recalled across the five consecutive trials, and the percentage delayed recall (AVLT DR), i.e. the score on the 7th trial divided by the score at the fifth trial times 100.

In the BSRT [31], instead of presenting all items repeatedly on each trial, only the items that have not been recalled on the immediately preceding trial are presented even though participants are instructed to recall the entire list of items on each trial. During this word-list learning test, 12 semantically and phonologically unrelated items are presented during 12 consecutive trials. From the BSRT, two performance parameters were derived: the total retention (BSRT TR) and the delayed recall (BSRT DR). BSRT TR is calculated by adding the scores of the 12 consecutive trials. When a participant recalls all 12 items on each of three consecutive trials, the test is prematurely concluded and a maximum score (i.e. a score of 12) is given for the following trials. The maximum total retention score over all 12 trials is 144 points. We used the average of the total retention score as a measure of word-list learning capacity. After 30 min, participants were asked to recall as many items as possible (BSRT DR, expressed as an absolute score (/12)).

Language and semantic processing were assessed with Boston Naming Test (BNT) [32], Animal Verbal Fluency (AVF), Letter Verbal Fluency (LVF) and the Psycholinguistic Assessment of Language Processing in Aphasia (PALPA) item 49, which probes verbal associative-semantic processing [33]. We measured executive functioning with the Standard Raven’s Progressive Matrices (RPM) [34] and Trial Making Test A and B (TMT), expressed as the ratio of score B over A.

Table 1 shows average ± SD cognitive tests scores for the total sample of 180 cognitively intact older adults.

The same tests were administered at every 2-yearly longitudinal follow-up visit for a total follow-up period of 10 years. The current report is based on the 4-year data. For the list learning tasks, the same versions were used for the successive testing sessions.

### Imaging

#### Structural magnetic resonance imaging

We acquired a high-resolution T1-weighted structural MRI using a 3D turbo field echo sequence on a 3 Tesla Philips Achieva system (Philips, Best, The Netherlands) (inversion time (TI)=900 ms, repetition time (TR)=9.6 ms, echo time (TE)=4.6 ms, flip angle = 8^∘^, field of view (FOV)=250 ×250 mm, 182 slices, voxel size 0.98 ×0.98×1.2 mm^3^). One subject was excluded from MRI due to safety reasons (occupation welder), yielding a total of 179 subjects with T1-weighted MRI.

Pre-processing of the T1-weighted MRI scans was performed with voxel-based morphometry 8 (VBM8, http://dbm.neuro.uni-jena.de/vbmrunning) using Statistical Parametric Mapping (SPM8, Wellcome Trust Centre for Neuroimaging, London, UK, http://www.fil.ion.ucl.ac.uk/spm) [35] in Matlab R2012b (Mathworks, Natick, USA). The resulting modulated grey matter volumes are adjusted for overall brain size by using the ‘nonlinear only’ component in the spatial normalization process for modulation of grey matter voxel intensities, as described in previous studies [36]. This procedure generated modulated grey matter volume maps (voxel size = 1.5 mm^3^) which were subsequently smoothed with an isotropic Gaussian kernel of 8 ×8×8 mm^3^ Full-width Half Maximum (FWHM) and masked with an absolute threshold of 0.1 in further voxelwise statistical analyses. Total intracranial volume (ICV) was extracted using the VBM toolbox with the individual’s segmentation parameters as input.

For region-based analyses, regional volumes were extracted by using the extended Brainnetome atlas [37] intersected with the individual’s unsmoothed modulated grey matter map. The median grey matter volume intensity was calculated for each subject and used in R statistical analyses described in subsequent paragraphs. The regions of main interest were the hippocampus (rostral (Brainnetome area 215, 216) and caudal (Brainnetome area 217, 218)) and entorhinal cortex (Brainnetome area 115, 116).

As an alternative approach, T1-weighted MRI scans were processed with FreeSurfer v6.0, for which cortical thickness as well as volumetric measures and ICV were automatically computed and extracted using the Desikan atlas [38]. FreeSurfer hippocampal and entorhinal volumes were corrected for ICV through the linear equation as used in [12, 39]: Vol _*adj*_ = Vol _*raw*_(i) − b(ICV(i) − Mean ICV), where Vol _*adj*_ is the adjusted volume, Vol _*raw*_(i) is the original volume for an individual, *b* is the slope of the volumes regressed on ICVs in the study sample, and mean ICV is the sample mean of ICV. ICV was divided by 1000 to be on approximately the same scale as the hippocampal/entorhinal volumes.

### ^18^F-Flutemetamol amyloid-PET imaging

All participants underwent ^18^F-flutemetamol PET on a 16-slice Biograph PET/CT scanner (Siemens, Erlangen, Germany). The tracer was injected as a bolus in an antecubital vein (mean activity 150 MBq, SD 5 MBq, range 134–162 MBq). Scan acquisition started 90 min after tracer injection and lasted for 30 min [27, 40]. Prior to PET acquisition, a low-dose CT scan of the head was performed for attenuation correction. Random and scatter correction were applied. Data were recorded in list mode and reconstructed into six 5-min frames using ordered subsets expectation maximization (four iterations × 16 subsets). Processing of ^18^F-flutemetamol PET was done using SPM8 running on Matlab R2012b as described in detail elsewhere [27]. A standardized uptake value ratio image (SUVR) was calculated using participant-specific cerebellar grey matter as a reference region (grey matter threshold: 0.3). Mean ^18^F-flutemetamol SUVR values were calculated for a composite region (SUVR _*comp*_), which consisted out of five bilateral cortical regions: frontal, parietal, anterior cingulate, precuneus-posterior cingulate and lateral temporal. These regions were derived from the Automated Anatomical Labeling (AAL) atlas [41] and intersected with the participant-specific grey matter, thresholded at 0.3. In a next step, SUVR _*comp*_ values were converted to Centiloids (CLs) [42] using as conversion formula CL = 127.6 × SUVR - 149. Low or absent amyloid burden was defined as CL ≤10 CL, intermediate amyloid burden as CL between 10 and 50 and high amyloid burden as CL ≥50 [43].

For whole-brain voxelwise analyses, ^18^F-flutemetamol SUVR images were smoothed with an isotropic Gaussian kernel of 8 ×8×8 mm^3^ FWHM.

Sixty-eight participants received ^18^F-flutemetamol PET prior to a major scanner upgrade (on 14/03/2012), while the remaining 112 received ^18^F-flutemetamol PET after the upgrade date. The scanner upgrade effect was included as a dummy variable in voxelwise statistical models.

### Statistical analyses

All standard statistical analyses were conducted with R statistical software version 3.6.2 (2019-12-12) (The R Foundation for Statistical Computing; cran.r-project.org). Test statistics are two-tailed and the significance threshold was set at an uncorrected *α* <0.05. Voxelwise statistical analyses were conducted in SPM8 software with the default significance threshold set at voxel-level uncorrected *P* value <0.001 combined with a cluster-level whole-brain Family Wise Error (FWE)-corrected threshold *P* value <0.05.

We assessed whether participants that had dropped out from the study at year four and retainees differed on baseline characteristics using Mann-Whitney *U* tests for continuous variables or Chi square statistics for categorical baseline variables.

#### Factor analysis

On the cross-sectional cognitive dataset, we performed a factor analysis using the R package *psych* (http://personality-project.org/r/psych/) on the following baseline cognitive scores of the 180 subjects: AVLT TL and DR, BSRT TR and DR, BNT, AVF, LVF, PALPA49, RPM, and the ratio of TMT subtest B over A. Prior to factor calculations, data imputation was applied in one subject for missing data on RPM and in another subject for missing data on BSRT DR using the R package *mice* (https://amices.org/mice/). To assess the suitability of the baseline cognitive dataset for factor analysis, we used the Kaiser-Meyer-Olkin (KMO) test for sampling adequacy (KMO value should be >0.600) as well as the Bartlett’s test for sphericity and correlational adequacy (*p* value should be <0.001). Only factors with an eigenvalue >1.0 according to the Kaiser’s criterion were retained [44]. The factors were rotated with a variance maximizing (varimax) orthogonal rotation to obtain interpretable and uncorrelated factor loadings. The goodness of fit of the factor model was assessed using the empirical chi-square statistic, Comparative Fit index (CFI) (>0.90) and the Root Mean Squared Error of Approximation (RMSEA) index (<0.10).

#### Latent growth curve analysis

In the primary analysis, the cognitive time course was modelled over a 4-year period by means of latent growth curve analysis of the cognitive test parameter that showed the highest factor loading. That parameter can be considered as most ‘representative’ for a given factor. The longitudinal scores for this test were modelled by means of latent growth curve analysis using the R package *lavaan* [45] (http://www.jstatsoft.org/v48/i02/). In this model, the growth factor consists of an intercept and a slope. The intercept is considered to be constant over time points while the slope is modelled as the linear change over time points. The slope of the cognitive test was computed using scores obtained at baseline, at 2 years and at 4 years of follow-up using the maximum likelihood default estimator in the *lavaan* package. A more negative slope implies a steeper decline. Missing values were imputed using the CART imputation method in *mice* as previously described. The goodness of fit of the latent growth model was assessed using the empirical chi-square statistic, with a lower value showing a better model fit (lowest chi-square statistic for the unconstrained model reported here = 2.85).

As a secondary analysis, we used Linear Mixed-Effects (LME) modelling to calculate the slope of cognitive decline instead of latent growth curve modelling. LME was applied to the longitudinal imputed dataset using the R package *lme4* (https://cran.r-project.org/web/packages/lme4) [46] with as input the cognitive test parameter that showed the highest loading for each factor.

As a further secondary analysis, instead of selecting the test with the highest factor loading for modelling cognitive change using latent growth curve analysis, a weighted composite factor score was calculated per cognitive domain and subjected to latent growth curve analysis. This approach requires factor weights: These were obtained from the factor analysis of the cross-sectional cognitive dataset [47, 48]. Prior to calculation of the weighted composite factor score, all neuropsychological tests were scaled using the scale function in R to a mean of 0 and standard deviation of 1. Subsequently, the Thurstone regression approach was applied to derive factor scores per cognitive domain, for which each predictor, i.e. the scaled neuropsychological test score, was multiplied by its corresponding factor weight [48]. Factor weights at baseline were constrained and used to derive weighted composite factor scores at 2 years and at 4 years of follow-up, similarly to [47]. These weighted composite factor scores were entered into a latent growth curve analysis in order to derive domain-specific factor slopes.

#### Whole-brain voxelwise regression analyses

The latent growth curve slope of 4-year cognitive change of the most representative test score for each factor was entered as regressor in a whole-brain voxelwise regression analysis with modulated grey matter as outcome variable. The 179 modulated grey matter volume images were entered as the dependent variable and the individual slope as the independent variable. ICV was added as covariate of no interest.

A whole-brain voxelwise regression analysis was also conducted with ^18^F-flutemetamol SUVR as outcome variable and the slope of the cognitive test score. PET scanner upgrade was added as a dummy variable. Statistics were calculated within an external mask created based on the full dataset of subjects, thresholded at 0.3 [27].

#### Correlation matrix

A correlation matrix was calculated between the slope of cognitive decline and age, sex, education, *APOE* and *BDNF* polymorphisms, amyloid-PET CL, and amyloid-PET SUVR in the precuneus. The slope was primarily calculated based on latent growth curve analysis of the most representative test parameter of each factor. In secondary analyses, we also calculated the slope based on LME and, separately, based on latent growth curve analysis of the weighted composite factor score, as described above.

Baseline cognitive test scores were included as predictors for slope of cognitive decline. To ensure independence between baseline test score and cognitive slope, in the primary analysis, the baseline measure was based on the test ranked second to the most representative cognitive test used for calculating the slope. In one of the secondary analyses, we included the weighted composite factor score at baseline as predictor.

Correlation with hippocampal volume and entorhinal volume and thickness was also determined. Visualization of the correlation matrix of data across the 179 participants was done using the R package *corrplot* (https://github.com/taiyun/corrplot).

#### Hierarchical regression analysis

Next, the variables that reached significance at an uncorrected *P* <0.05 in the correlation matrix were entered into a hierarchical regression analysis. The R package *caret* was used to compute repeated k-fold cross-validated models (http://topepo.github.io/caret/index.html). To this end, the dataset was first scaled by the scale function in R to obtain interpretable beta coefficients and then randomly split into k-subsets (i.e. k-fold). The parameter *k* was set at 10 with three repeats.

The base model consisted of age and sex. Next, the other variables were added one by one according to the strength of the correlation (first the variable showing the strongest correlation coefficient and so forth). We determined whether the Root Mean Squared Error (RMSE) decreased and *R*-squared based on resampling increased by adding variables. In each step, the models were compared pairwise so that the regression model that produced the lowest test sample RMSE and highest *R*-squared was the preferred model.

We evaluated the influence of the statistical method used to calculate the baseline cognitive state and the slope of cognitive decline. Separate *k*-fold cross-validated regression models were fit using the LME-based slope as dependent variable, or the weighted composite factor slope as dependent variable, or using as predictor the baseline weighted composite factor scores.

As an additional analysis, we subdivided the dataset based upon amyloid status (CL cutoff = 23.4) [49] and assessed within amyloid-positive and amyloid-negative groups how age, baseline episodic memory (i.e. AVLT TL score) and MRI measures relate to the slope of cognitive decline.

#### Mediation analysis

It has been hypothesized that reduced episodic memory with age is mediated by hippocampal volume loss [18, 22] and that the hippocampal volume loss is partly a consequence of increased brain amyloidosis [23]. We tested these hypotheses by means of mediation analysis. Mediation analysis was performed using the R package *lavaan* [45]. Only variables were included in the mediation analysis that correlated significantly with episodic memory: In the absence of a correlation, no direct or indirect effect can be expected in mediation analysis. Two serial mediation analyses were computed with age as predictor variable and BSRT slope as outcome variable. In the first mediation model, we tested the hypothesis that the relation between age and episodic memory decline was mediated via hippocampal volume loss [18, 22]: The serial mediators between age and BSRT slope were hippocampal volume and AVLT TL baseline scores. In the second mediation model, we tested the hypothesis that amyloid load mediated the relationship between age, hippocampal volume, and episodic memory decline, the serial mediators consisted of precuneus SUVR and hippocampal volume. Using a simple mediation model, we assessed wether hippocampal volume mediated the relationship between amyloid load and cross-sectional episodic memory, using baseline BSRT scores [23]. Path-weights were displayed as *β* coefficients with corresponding standard errors. Significance of the indirect effect was determined using a nonparametric bootstrap method (10,000 iterations) [50]. The significance threshold was set at an uncorrected *α* <0.05.

## Results

**Fig. 1:**
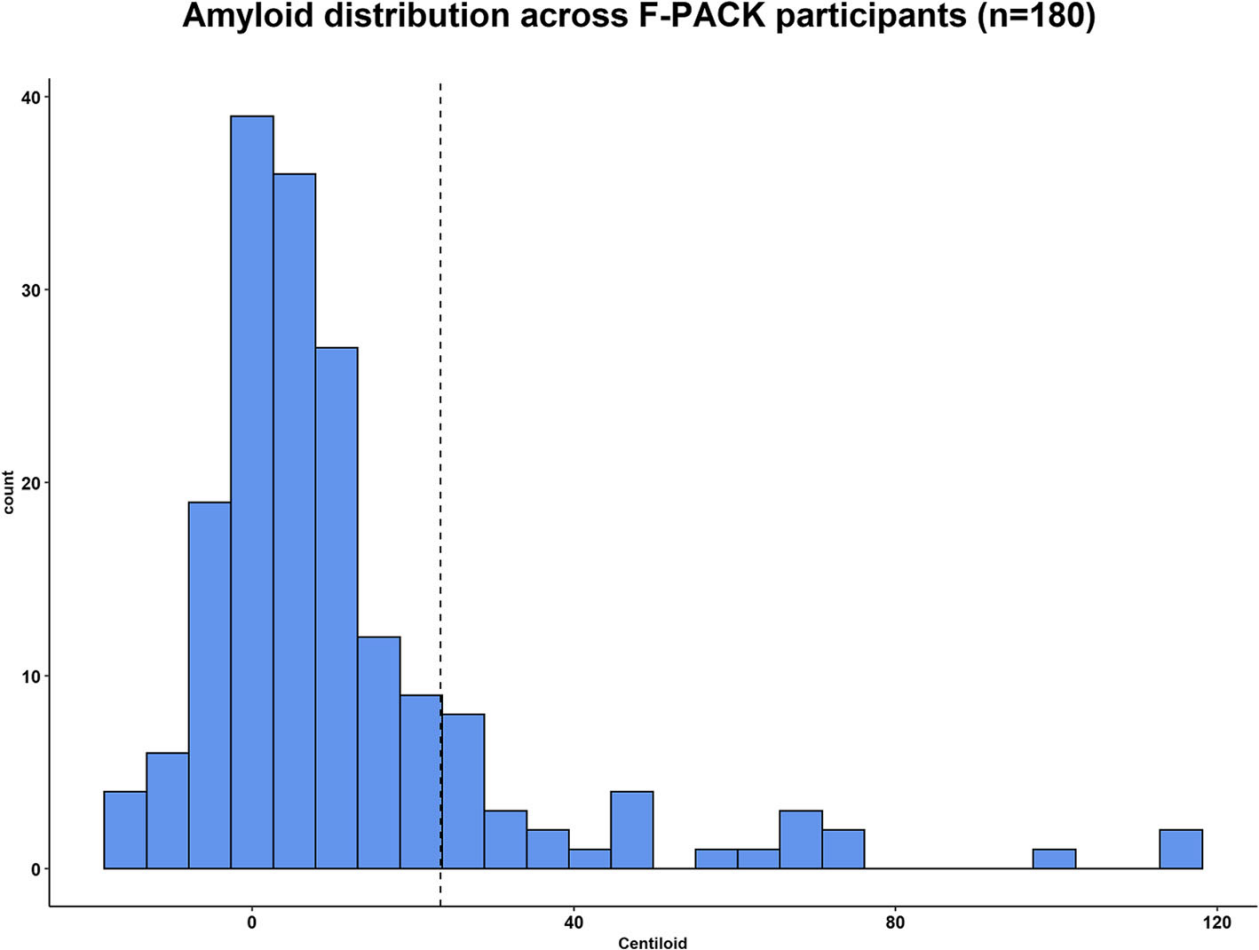
Histogram of baseline amyloid burden across F-PACK participants. Centiloid values of the total cohort of 180 participants are plotted as index for amyloid load. Histogram binwidth = 5.25. Dashed vertical line = neuropathologically validated CL cutoff of 23.43 for binary stratification of the cohort into amyloid-positive and amyloid-negative cases

Of the 180 cognitively intact older adults, 112 (62%) showed no amyloid burden (CL range −16.8 to 9.6), 58 (32%) low to intermediate (CL range 10.3–47.8) and 10 (6%) high amyloid burden (CL range 59.2–116.6) [43] (Fig. 1). Based on a neuropathologically validated binary cutoff of 23.4 CLs [49], 28 participants (15.6%) were amyloid-positive and 152 (84.4%) were amyloid-negative.

At year four, 25 individuals (13.9%) had dropped out from the study. Participants who dropped out were significantly older at baseline (dropouts: mean ± SD = 71.1 ±4.9; retainees: 68.2 ±6.6, *P* = 0.036), had lower MMSE (dropouts: 28.7 ±1.0; retainees = 29.1 ±0.9, *P* = 0.044), lower AVLT TL scores (dropouts: 42.6 ±9.6; retainees = 47.9 ±8.9, *P* = 0.012) and lower RPM scores than retainees (dropouts: 39.2 ±7.8; retainees = 42.2 ±8.8, *P* = 0.027). Other baseline variables such as amyloid load or genetic polymorphisms did not differ between drop-outs and continuers (all *P* >0.065).

### Factor analysis of neuropsychological test scores

The KMO test for sampling adequacy (KMO value = 0.73) and Bartlett’s test for sphericity (*P* = 3.48e ^−74^) confirmed the suitability of the baseline cognitive dataset for factor analysis. Factor analysis of the cognitive dataset resulted in two factors explaining a total of 39% of the variance in the baseline cognitive scores (Table 2). The first factor grouped measures of verbal episodic memory. The parameter with the highest standardized factor loading was the BSRT TR; hence, this parameter will be considered most representative for the first factor and used for modelling the slope in episodic memory (see below). The second factor contained the semantic and phonological verbal fluency tasks and confrontation naming as tests with highest loading. The AVF had the highest standardized factor loading on the second factor and will be considered most representative and used for modelling the verbal fluency slope.

**Table 2:** Factor analysis of the baseline neuropsychological test results

	Factor 1	Factor 2
Eigenvalue	2.14	1.79
Variance explained (%)	21.0	18.0
Cumulative variance explained (%)	21.0	39.0
BSRT TR	0.98	0.12
BSRT DR	0.79	0.11
AVF	0.04	0.65
LVF	0.16	0.62
AVLT TL	0.59	0.32
BNT	0.01	0.58
RPM	0.30	0.47
AVLT %DR	0.28	−0.03
PALPA49	−0.05	0.41
TMT B/A	−0.13	−0.35

First column: Tests. Column 2–3: Standardized factor loadings. Loadings greater than 0.7 are marked in bold. Abbreviations: For abbreviations, see Table 1. Model fit parameters were within recommended norms: empirical chi-square 29.04, *P* <0.31, Comparative Fit index = 0.98 (>0.9) and RMSEA index = 0.045

### Whole-brain voxelwise regression analysis with cognitive slope

**Fig. 2:**
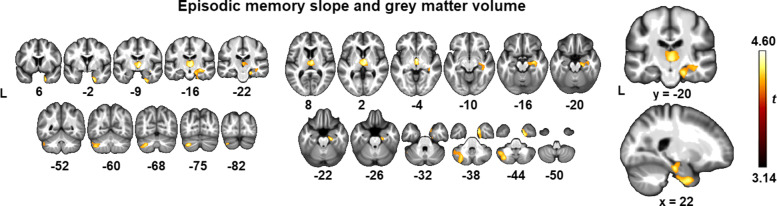
Whole-brain voxelwise regression analysis of the individual modulated grey matter maps. The regression included individual grey matter maps as dependent variable and episodic memory slope, i.e. Buschke Selective Reminding Test total retention slope, as independent variable. The one-sided *t*-contrast is overlaid on sections of the average bias-corrected MNI normalized T1 MRI of the 179 subjects (brighter colour depicts higher *t* value). The significance threshold was set at voxel-level uncorrected *P* value <0.001, with a cluster-level whole-brain FWE-corrected threshold *P* value <0.05

To model the change in episodic memory, the slope of the BSRT TR over a 4-year time course was determined by means of latent growth curve analysis (in later sections referred to as the *episodic memory slope*). Next, this slope was entered in a whole-brain voxelwise regression analysis with modulated grey matter volume. Significant clusters at a cluster-level whole-brain FWE-corrected threshold *P* value <0.05 (with voxel-level set at uncorrected *P* <0.001) were located in the right medial temporal cortex, mainly the entorhinal cortex, parahippocampal gyrus and hippocampus, as well as in the dorsomedial thalamus and in the left cerebellar crus II (Fig. 2, Table 3). At a lower threshold of voxel-level uncorrected *P* value<0.001, there was an additional cluster in the left medial temporal lobe including the entorhinal cortex, parahippocampal gyrus and hippocampus.

**Table 3:** Whole-brain linear regression analysis of episodic memory decline neural correlates

Region	MNI coordinates			Extent	*P*_*FWE*_	*Z*
	*x*	*y*	*z*			
Right medial temporal cortex	20	2	−39	1686	0.002	4.03
	21	−19	−23			3.98
	32	−15	−15			3.97
Dorsomedial thalamus	6	−9	1	1402	0.006	4.46
	3	−18	−2			4.31
Left cerebellar crus II	−30	−72	−42	1360	0.007	3.99
	−41	−60	−45			3.74

Peak coordinates of the whole-brain voxelwise linear regression analyses across 179 subjects with the individual modulated grey matter maps as dependent variable, and Buschke Selective Reminding Test total retention slopes as index for episodic memory decline as independent variable. ICV is used as covariate of no interest. The significance threshold was set at voxel-level uncorrected *P* value <0.001, with a cluster-level whole-brain FWE-corrected threshold *P* value <0.05. MNI coordinates (*x*, *y* and *z*) are in mm. Extent corresponds to the number of grey matter voxels within a cluster, of which the voxel size was 1.5 mm^3^

A same procedure was applied for the verbal fluency slope: The whole-brain voxelwise regression analysis did not reveal significant clusters.

Whole-brain voxelwise regression analyses with ^18^F-flutemetamol SUVR images as dependent variable did not yield significant clusters for either the episodic memory slope or the verbal fluency slope at the preset significance threshold. At a lower threshold of voxel-level uncorrected *P* value<0.001, steeper episodic memory decline correlated with increased amyloid in the precuneus.

### Predictors of cognitive decline

**Fig. 3:**
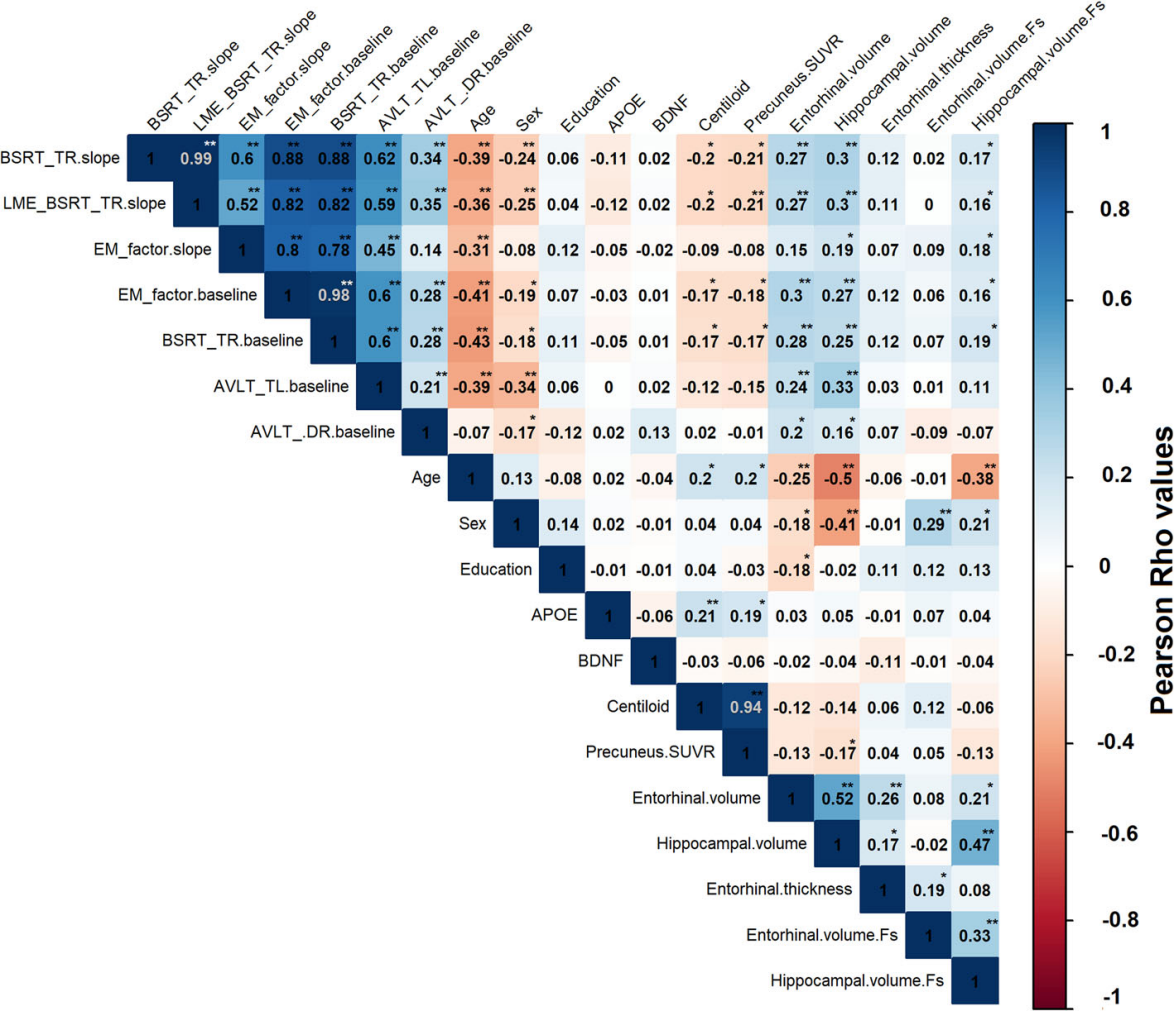
Correlation matrix for episodic memory decline. Correlation matrix assessing the relation between cognitive decline on the BSRT TR score, weighted composite episodic memory factor and other variables. The colour scale depicts the strength of the Pearson correlation coefficient (*Rho*). A more positive value for the cognitive slope corresponds to less decline. Superimposed text reflects the actual numerical value of the Pearson correlation coefficient. The last three cells are FreeSurfer-based imaging measures: cortical thickness as specified, as well as ICV adjusted grey matter volumes (.Fs). The asterisk depicts the significance of the correlation: **P value <0.001, *P value <0.05. *APOE*, Apolipoprotein E; AVLT, Rey Auditory Verbal Learning Test; TL, total learning; DR, long-term percentage delayed recall; *BDNF*, Brain-Derived Neurotrophic Factor; BSRT TR, Buschke Selective Reminding Test total retention; EM, episodic memory; LME, linear mixed-effects; SUVR, standardized uptake value ratio image

In order to gain a better understanding of the association between the different baseline variables and the slope of episodic memory decline, a correlation matrix was calculated (Fig. 3). As baseline cognitive test for prediction, the AVLT TL was chosen as it ranked highest on the first factor behind the BSRT TR and BSRT DR, so as to avoid dependence between the test for prediction and the test for modelling the slopes of cognitive decline.

**Fig. 4:**
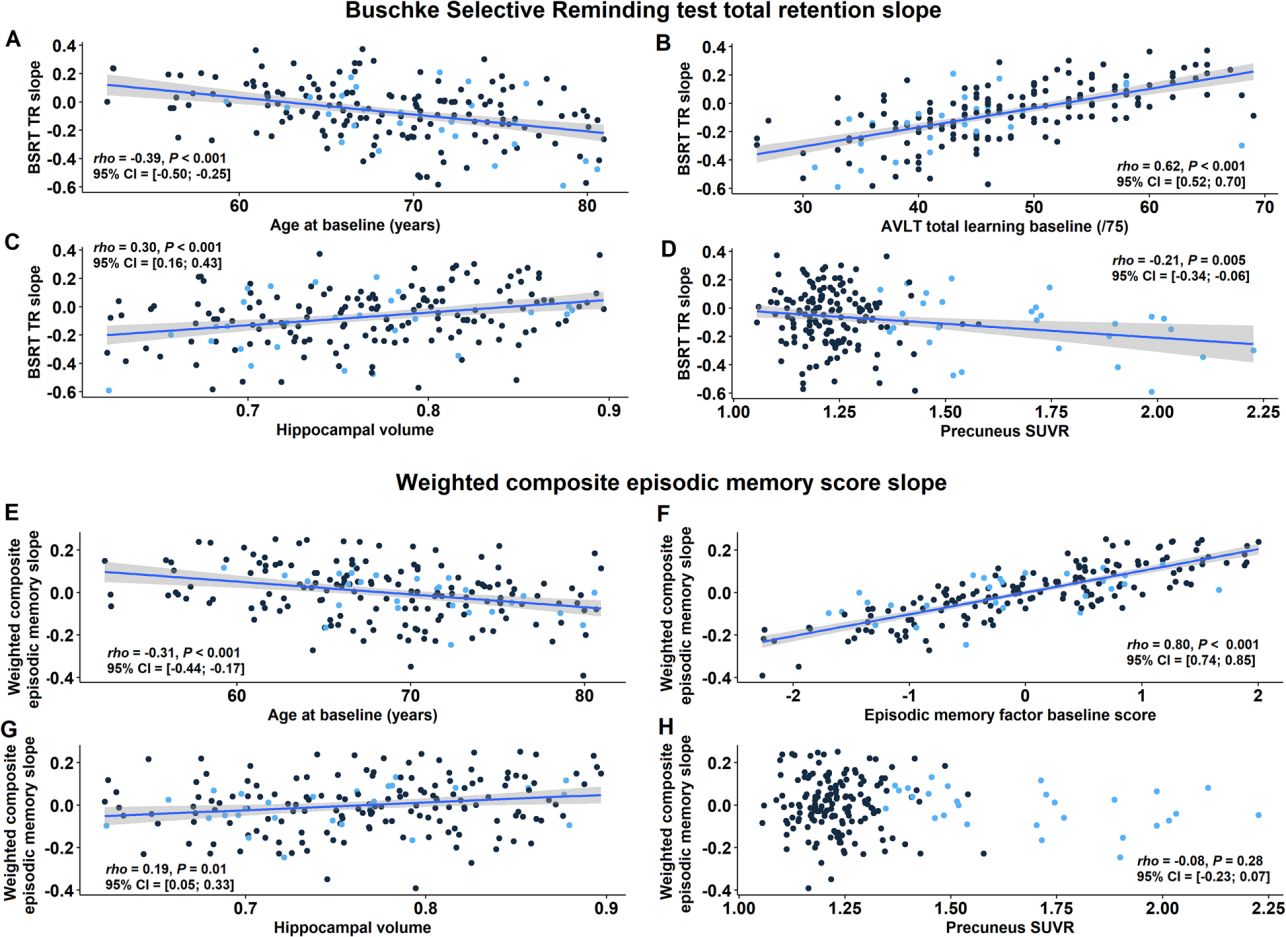
Predictors of episodic memory decline. A more positive value for BSRT TR slope corresponds to less decline. Correlation between episodic memory slope and **a** age at baseline, **b** Rey Auditory Verbal Learning Test (AVLT) total learning at baseline, **c** VBM hippocampal grey matter volume and **d** precuneus SUVR as index for amyloid load. Correlation between weighted composite episodic memory factor slope and **e** age at baseline, **f** episodic memory factor score at baseline, **g** VBM hippocampal grey matter volume and **h** precuneus SUVR. Data points have been colour-coded based upon amyloid status (CL cutoff = 23.4): *n* = 152 amyloid-negatives (black), *n* = 28 amyloid-positives (blue). Note that for all variables the sample size is *n* = 180, except for hippocampal volumes: *n* = 179; Significant associations are plotted as least squares regression line ±95% confidence interval; Pearson *rho*. AVLT, Rey Auditory Verbal Learning Test; BSRT TR, Buschke Selective Reminding Test total retention; SUVR, standardized uptake value ratio image

The episodic memory slope, i.e. BSRT slope, correlated negatively with age (Pearson corr. = −0.39, *P* <0.001): Higher age was associated with a steeper negative slope (i.e. more episodic memory decline) (Fig. 4a). The episodic memory slope correlated strongly with the baseline AVLT TL score (Pearson corr. = 0.62, *P* <0.001): The lower the baseline AVLT score, the more negative the slope, hence the steeper the episodic memory decline (Fig. 4b). Regarding volumetric MRI measures, there were also significant correlations between the episodic memory slope and hippocampal volume (Pearson corr. = 0.30, *P* <0.001) (Fig. 4c) as well as with entorhinal volume (Pearson corr. = 0.27, *P* <0.001). The episodic memory slope also correlated inversely with amyloid CL values (Pearson corr. = -0.20, *P* = 0.01) and precuneus SUVR (Pearson corr. = −0.21, *P* = 0.01) (Fig. 4d). Furthermore, there was a significant correlation between precuneus SUVR values (i.e. index for early amyloid burden) and hippocampal volume (Pearson corr. = -0.17, *P* = 0.03) (Fig. 3). There were no significant effects of genetic *APOE* or *BDNF* polymorphisms on the episodic memory slope.

When the slope of cognitive change was based on LME rather than latent growth curve analysis, results were entirely comparable, as one would expect given the very high correlation between the two slopes (Pearson corr. = 0.99, Fig. 3). When the slope was based on latent growth curve analysis of weighted composite episodic memory factor scores, significant correlations were found between this slope and age (Pearson corr. = -0.31, *P* <0.001) (Figs. 3 and 4e), baseline episodic memory (measured either as baseline weighted composite score (Pearson corr. = 0.80, *P* <0.001) (Fig. 4f), BSRT TR (Pearson corr. = 0.78) or AVLT TL) (Pearson corr. = 0.45) and hippocampal volume (Pearson corr. = 0.19, *P* = 0.01) (Fig. 4g). Overall, correlations were always lower for the weighted composite slope than for the BSRT TR slope (Fig. 3). The correlation between the weighted composite slope and the entorhinal volume did not reach significance (Pearson corr. = 0.15, *P* = 0.05), and neither was there a correlation between baseline amyloid load and the slope of the weighted composite episodic memory factor decline (Pearson corr. = −0.08, *P* = 0.28) (Fig. 4h).

The verbal fluency slope only correlated with age (Pearson corr. = −0.20, *P* = 0.01).

#### Hierarchical regression

Each variable that showed a significant correlation with the episodic memory slope in the simple regression analyses at *P* ≺0.05 uncorrected (Fig. 3) was entered into the hierarchical regression analysis in the following order: First, a baseline model was tested with age and sex as only predictor variables: This model (F(2, 176) = 20.1, *P* <0.001) accounted for 18.5% of the variance (*R*^2^) in episodic memory slope. Addition of the AVLT TL baseline scores increased this to 41.6%. In the latter model (F(3, 175) = 39.9, *P* <0.001), AVLT TL baseline scores (*β* = 0.54 (SE = 0.07), *P* <0.001) and age (*β* = −0.17 (SE = 0.06), *P* = 0.007) had a significant effect while the effect of sex was no longer significant (*P* = 0.53). Adding any of the imaging variables hardly increased this any further (maximum 42.2% for a model with age, sex, AVLT TL baseline and entorhinal volume).

The strong effect of an individual’s baseline episodic memory performance did not depend on the order of variables entered into the model: When hippocampal volume was added to the baseline model (age and sex), the variance explained (*R*^2^) by this model (F(3, 175) = 13.6, *P* <0.001) was reduced with 0.5%. When we added AVLT TL baseline scores to the latter model (age, sex, hippocampal volume), *R*^2^ increased with 23.0% (F(4, 174) = 29.9, *P* <0.001). This demonstrates the explanatory power of baseline cognitive scores, regardless of the order in which imaging and cognitive variables were entered into the regression model.

Similar results were obtained with the LME-based BSRT slope: a base model of age and sex explained 19.9% of variance, and addition of baseline memory scores increased the variance to 38.6% with a minimal increase to 38.8% when biomarker measures were added. When weighted composite episodic memory factor slopes were used to model cognitive change, the base model explained 12.6% of variance. This increased to 65.8% by adding the baseline weighted composite episodic memory factor scores. The value is higher than for the primary analysis using BSRT TR slope and AVLT TL as predictor but this can be attributed to the overlap in test parameter selection for baseline and slope when composite factor scores are used for baseline as well as slope modelling. Addition of biomarker measures increased the explained variance to 65.96%. These findings confirm that the results are independent from the exact statistical methods used to calculate cognitive decline or baseline cognitive performance.

Taken together, apart from age, by far the strongest predictor for the episodic memory slope was the baseline episodic memory score for all methods, nearly obliterating any additional effect of the volumetric MRI measures.

**Fig. 5:**
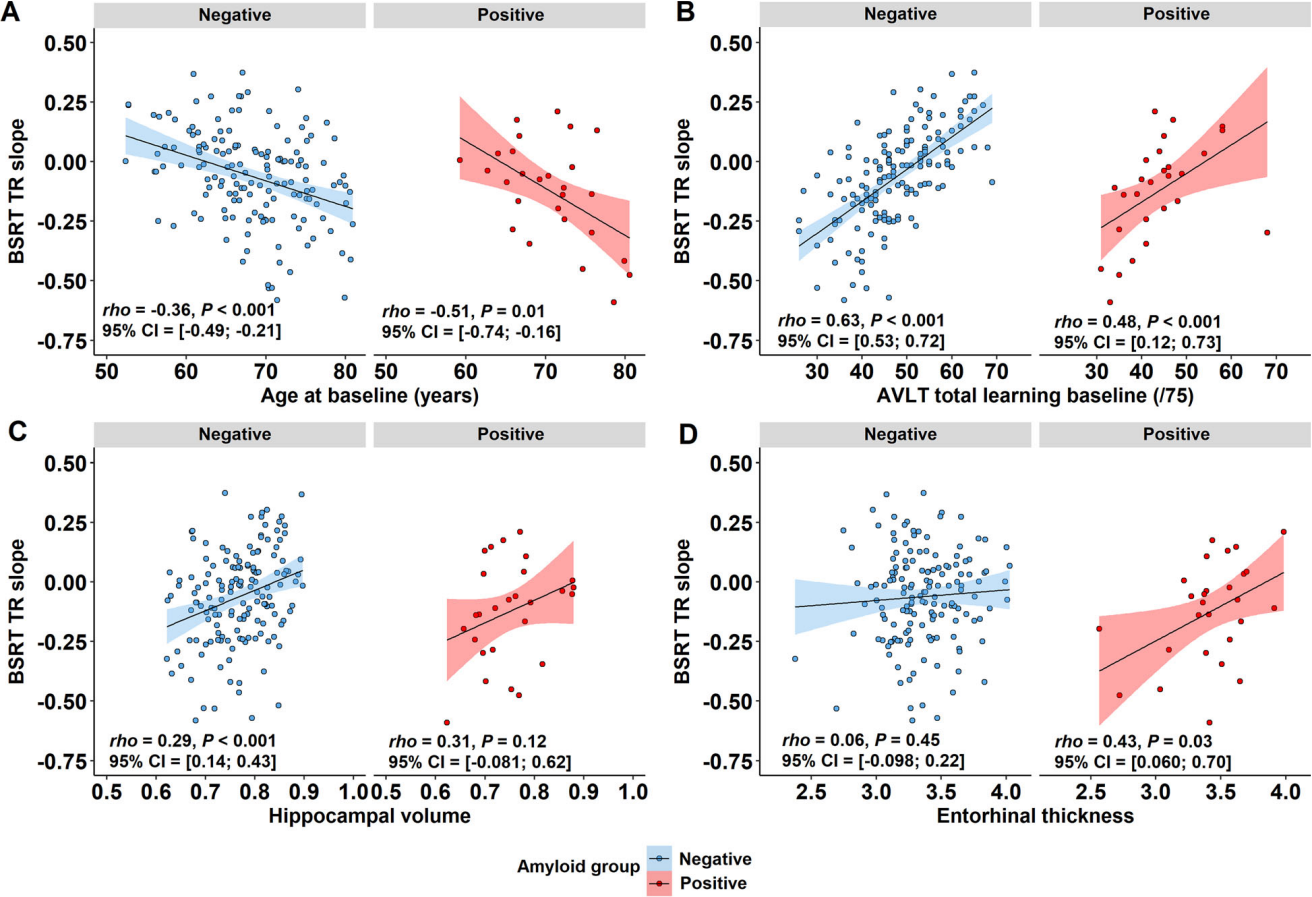
Regression analysis in the amyloid-positive and the amyloid-negative subgroups. Correlation between episodic memory slope and **a** age at baseline, **b** Rey Auditory Verbal Learning Test (AVLT) total learning at baseline, **c** VBM hippocampal grey matter volume and **d** entorhinal thickness. Correlational plots have been divided based upon amyloid status (CL cutoff = 23.4): *n* = 152 amyloid-negatives, *n* = 27 amyloid-positives; Pearson *Rho* and the corresponding uncorrected *P* value are plotted per amyloid group. Association illustrated as least squares regression line ±95% confidence interval. Abbreviations: AVLT, Rey Auditory Verbal Learning Test; BSRT TR, Buschke Selective Reminding Test total retention

When we subdivided the dataset into two subgroups based upon amyloid status (amyloid-negative versus amyloid-positive, CL cutoff = 23.4 [49]), the correlation of age and AVLT TL baseline score, respectively, with BSRT TR slope was present in each of the two subgroups (in amyloid-positives: Pearson corr. with age = −0.51, *P* = 0.01, with baseline scores = 0.48, *P* <0.001; in amyloid-negatives: Pearson corr. with age = −0.36, *P* <0.001, with baseline scores = 0.63, *P* <0.001). More specifically, within the amyloid-positive group, a model consisting of age and AVLT TL baseline scores as explanatory variables explained 75.6% of the variance (*R*^2^) in episodic memory slope. Adding precuneus SUVR values or entorhinal thickness to the model, icreased this to 76.2% and 79.4%, respectively. In the amyloid-negative group, a model consisting of age and AVLT TL baseline scores as explanatory variables explained 44.1% of variance in episodic memory slope, with no additional effect of the volumetric measures or amyloid load (Fig. 5).

#### Mediation analysis

**Fig. 6:**
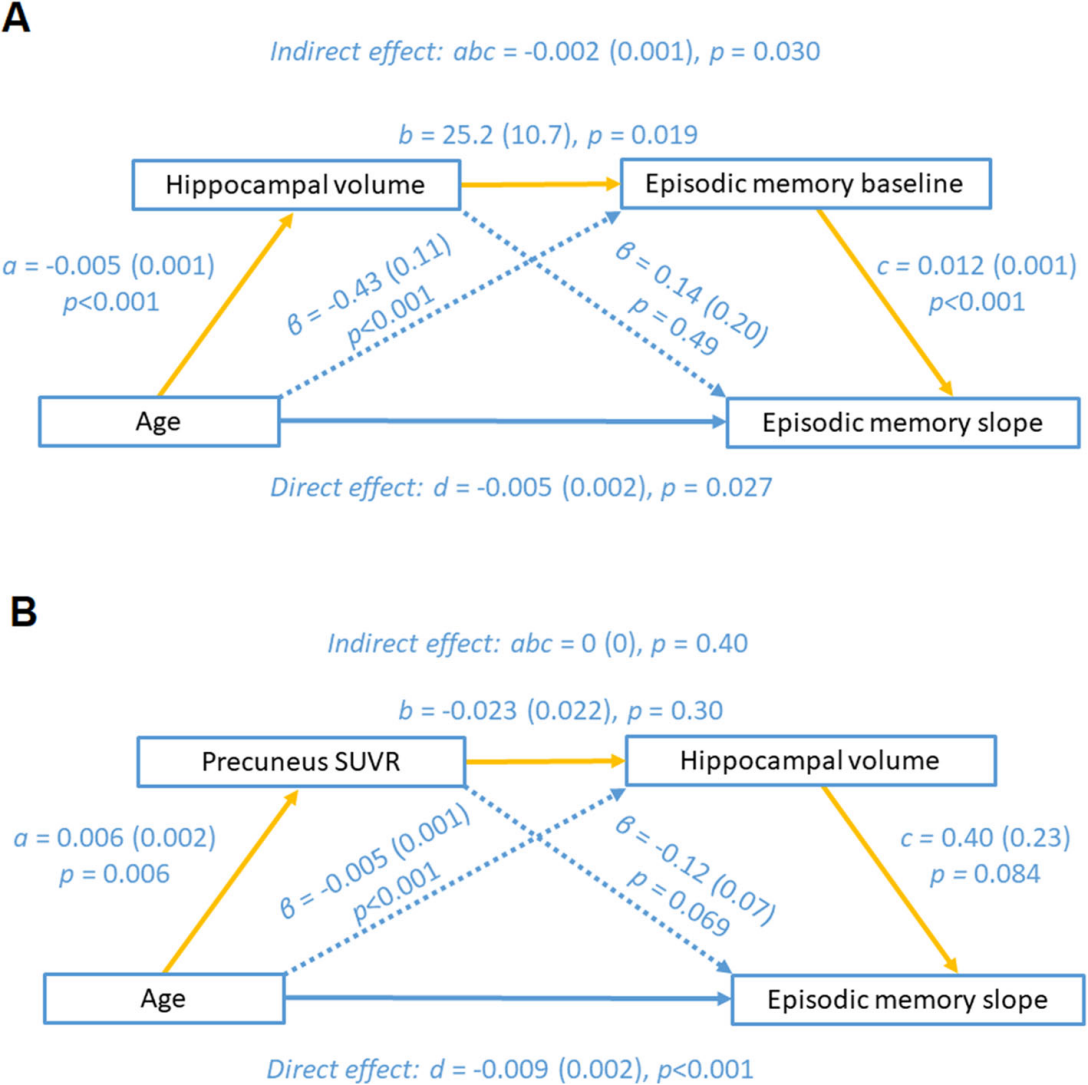
Mediation models. Results of the serial mediation analyses with episodic memory slope, i.e. BSRT slope as outcome variable. **a** The first mediation model contains age as predictor and as serial mediators: hippocampal volume and AVLT TL scores for baseline episodic memory performance. **b** The second mediation model contains age as predictor and as serial mediators: precuneus SUVR as index for amyloid load as well as hippocampal volume. Path-weights are displayed as *β* coefficients with standard errors between brackets. Significance of the indirect effect was determined using bootstrapping with 10,000 iterations. SUVR, standardized uptake value ratio image

We tested two hypotheses by means of serial mediation analysis. The first hypothesis [18, 22] stated that the effect of age on episodic memory scores was mediated via hippocampal volume loss. A causal model was constructed with edges from age to hippocampal volume to baseline episodic memory score to episodic memory slope, together with a direct path from age to baseline episodic memory score (Fig. 6a). The path model of this mediation analysis is shown in Fig. 6. The direct path from age to episodic memory slope was significant, as was the indirect path via baseline episodic memory scores: This means that part of the variance in episodic memory slope that is explained by age is not mediated via hippocampal volume. However, the indirect path from age through hippocampal volume to baseline episodic memory to episodic memory slope was significant (*abc* = −0.002, *P* =0.030). The latter effect confirms the a priori hypothesis [18, 22] (Fig. 6a).

In the second hypothesis, a mediating role of amyloid load was added to the model. We refuted this hypothesis: No serial mediation from amyloid load to hippocampal volume to episodic memory slope was present (*abc* = 0, *P* =0.40) (Fig. 6b). When we replaced episodic memory slopy with baseline episodic memory scores, no serial mediation effect of amyloid load and hippocampal volume could be obtained either (*abc* = 0, *P* =0.63). Interestingly, when comparing the latter two models, higher amyloid load showed a nearly significant association with steeper episodic memory slope (*β* = −0.12 (SE = −0.068), *P* = 0.069), but not with lower baseline memory scores (*β* = −0.56 (SE = 0.44), *P* = 0.20). Using a simple mediation model, we showed that there was no mediation effect of hippocampal volume on the relationship between amyloid load and baseline BSRT scores in our study sample (*ab* = −0.24, *P* =0.067) [23].

## Discussion

In cognitively intact older adults, the best predictor of 4-year episodic memory decline, besides age, are the baseline episodic memory scores, with only very limited additional variance explained by imaging measures of medial temporal grey matter volume or amyloid load. Second, the effect of age on episodic memory scores at baseline and on cognitive decline was only partly mediated via medial temporal grey matter volume loss, without mediating effect or amyloid load. In that sense, the data can be seen as a warning against a ‘biomarkers first’ motto even in the cognitively intact population.

### Baseline cognitive scores as predictor

The memory test with the highest loading on the episodic memory factor was the BSRT TR. This test captured the variability in the cognitively normal older population in a better manner than the AVLT, which is conventionally used in the clinic to discriminate normal from pathological episodic memory performance. A number of our subjects spontaneously reported that they found the BSRT more demanding than the AVLT while the inverse was never mentioned. This may be related to the fact that the word list is not repeated in its entirety at each trial and hence words are repeated less frequently. It may also relate to the presence of some abstract words in the BSRT list but not in the AVLT list [51]. In any case, the higher loadings for BSRT parameters than AVLT parameters on the first factor indicates higher variance accounted for by the BSRT parameters in this cognitively normal group and hence a higher suitability of BSRT for assessing episodic memory variability in cognitively normal individuals. The BSRT differs from its derivative, the Free and Cued Selective Reminding test (FCSRT) [53], where category cues are provided during both encoding and retrieval. These category cues are assumed to result in a more pure measure of ‘genuine memory’ (as opposed to ‘apparent memory’) that is less affected by differences in cognitive control during encoding or retrieval [6, 52, 53]. For use in a cohort of cognitively normal older adults, we opted for the BSRT as it can be expected to be more demanding to the participants given the higher need for strategic control during encoding and retrieval.

Factor analysis revealed that episodic memory test scores explained the largest amount of variance in the neuropsychological dataset. BSRT TR loaded most heavily on this first factor with highest explanatory power. Hence, it was considered the most representative test for factor 1 and used to model the slope of episodic memory decline. In order to avoid dependency between predictor and slope, we did not use BSRT parameters as predictors but used the second highest ranked test in the first factor, obtained from the factor analysis, i.e. AVLT TL. The sensitivity of AVLT TL for predicting change is in line with the Baltimore Longitudinal Study of Aging [54]. In another study in cognitively healthy older adults, the total learning on the California Verbal Learning Test and the Selective Reminding test predicted conversion from a CDR of 0 to a CDR of 0.5 over an 11-year time period [55].

In a recent study including cognitively healthy individuals as well as patients who were cognitively impaired (lowest MMSE = 16) [56], the strongest predictor for cognitive decline (measured based on MMSE and CDR) were baseline episodic memory scores (Wechsler Memory Scale Logical Memory test) rather than MRI volumetric measures. The current findings confirm the predictive power of baseline cognitive scores in predicting cognitive decline in cognitively intact older adults, outweighing amyloid or volumetric MRI measures.

How can the strong effect of baseline episodic memory score on the slope of decline be explained? Baseline episodic memory scores are a snapshot that reflects the integral of the individual’s initial peak level of performance (probably situated decades before study inclusion) together with the individual’s subsequent cognitive trajectory. Evidently, for a same initial peak performance level, an individual who scores in the low normal range at study baseline will have had a steeper downward trajectory in the years before study inclusion. Accordingly, this individual will also be more likely to continue on this downward trajectory. Alternatively, one could interpret the low baseline score as a sign of low cognitive reserve which in its turn could be predictive of more rapid cognitive decline. The latter hypothesis is less likely as we did not find a correlation with educational level.

### Age, medial temporal volume and episodic memory

In a simple regression, there was a clear association between hippocampal volume and subsequent cognitive decline in cognitively intact individuals, in line with previous studies [9]. Based on the Betula Prospective Cohort [18, 22], the medial temporal lobe system has been proposed to be the primary pathway of episodic-memory decline in normal, nondemented ageing. We tested this hypothesis by means of a mediation analysis. The effect of age on episodic memory slope was indeed partly mediated via hippocampal volume and baseline episodic memory scores. However, there remained also a direct effect from age to episodic memory that was not mediated by hippocampal volume. In a number of previous cross-sectional analyses [20, 57], the correlation between hippocampal volume and episodic memory disappeared when correcting for age. Hence, other factors must contribute to the relation between age and episodic memory decline than medial temporal lobe volume. These could consist of age-related hippocampal alterations in the hilus of the dentate gyrus and the subiculum below the MRI detection threshold [58] or rely on non-hippocampal pathways. Rather than hippocampal volume loss, changes in functional or structural connectivity [59] may account for the variance in episodic memory in older adults [60]. Changes in connectivity within the default mode network (DMN) predict changes in memory performance, whereas atrophy of the corresponding grey matter and hippocampal volume does not [5]. Changes in structural connectivity of frontostriatal circuits, the cingulum and the anterior portion of the corpus callosum have also been associated with age-related memory loss [22, 59].

Two previous studies have applied mediation analysis to examine the link between hippocampal volume and episodic memory during normal cognitive ageing. In an early study [61], the mediators consisted of a relatively extensive series of regional volumes (including, among others, hippocampal and lateral prefrontal volume) and cognitive variables (such as processing speed and working memory [3]). Once all cognitive and neuroanatomical mediators were taken into account, no direct effect of age on episodic memory remained. In another study [62] greater hippocampal volume (31.5% of age-related variance) and greater DMN connectivity (7.3% of age-related variance) both contributed significant independent variance to memory performance. The path coefficient from age to hippocampal volume was −0.45 and from hippocampal volume to memory 0.32. However, age group remained a significant predictor of memory after accounting for each imaging marker, in line with what we found [62].

### Amyloid, episodic memory and age

An effect was observed of baseline amyloid load on episodic memory decline, confirming earlier studies [9, 10, 13, 14, 63]. The size of the effect of baseline amyloid load on episodic memory decline was relatively small, as in previous studies [64]: a mean difference of 0.56 points over 4 years on the MMSE and 0.23 points on the CDR-Sum of the Boxes [10]. According to the Harvard Aging Brain Study (HABS), the survival curves start to diverge only 4.5 years after baseline [11].

Our findings are not compatible with a hypothesis that amyloid drives episodic memory decline in cognitively intact older adults through the mediating effect of hippocampal atrophy [23]. In the F-PACK cohort, the proportion of amyloid-positive cases is 15%, which is lower than in the cohorts studied by [23]. If a cohort contains a relatively high proportion of older adults in a preclinical AD stage, amyloid load, hippocampal volume and episodic memory decline are more likely to show a correlation as one would expect in AD.

### Genetic risk factors and episodic memory decline

We did not find an effect of *APOE* *ε*4 on the rate of episodic memory decline in this cognitively intact cohort. In the Longitudinal Aging Study Amsterdam, there was also no effect of *APOE* *ε*4 on 6-year change in delayed recall on the AVLT [65]. In another study [55], *APOE* *ε*2 was associated with a lower rate of conversion from CDR 0 to 0.5 in cognitively intact individuals, without any effect of *APOE* *ε*4 in this group. Other studies however showed an effect of *APOE* *ε*4 on the rate of cognitive decline in baseline cognitively intact individuals from the age of 60 onwards [66, 67]. If amyloid biomarker status is taken into account, the effect of *APOE* *ε*4 on cognitive decline in cognitively intact older adults was seen only in the amyloid-positive subgroup [68]. Taken together, these findings suggest that the effect of *APOE* *ε*4 on episodic memory decline in healthy older adults is linked to amyloid and preclinical AD.

### Study implications

With the advent of amyloid and tau biomarkers, there is a move towards more and more reliance on biomarkers to characterize and diagnose individuals and predict the future course. For instance, the A/T/N scheme [69] has rapidly become popular in AD research circles. The current study highlights the value of relatively inexpensive and broadly available cognitive assessment. The predictive value of cognitive scores outperformed that of amyloid PET and structural MRI measures in a very clear manner. The current findings warn against a biomarker-exclusive approach to case classification in cognitively intact individuals and highlight the value of cognitive assessment even in an era where much more expensive and high-tech options are available. This has practical implications: The A/T/N scheme does not contain a cognitive dimension and assumes that the cognitive performance level is the explanandum (what needs to be explained). The current study demonstrates that cognitive performance level contains a huge amount of hidden variables that are not captured adequately by the amyloid, tau and neurodegeneration scheme, let alone by a binary division based on these variables. These hidden variables may relate to cognitive reserve, functional brain circuitry, and many other brain variables that underlie cognition. In other words, cognitive performance is a summary measure that cannot be substituted by a simplified binary three-dimensional scheme and comprises effects of pathophysiological mechanisms that are missing from the A/T/N scheme.

### Study limitations

As subjects entered the study between 2009 and 2015, the multimodal assessment at baseline did not include tau PET imaging. This may be an imaging variable with higher 4-year predictive value, possibly at a level more comparable to baseline scores. A longitudinal imaging study examined how changes in amyloid predict changes in the spread of tau on PET and cognitive decline [11]. Increase in amyloid predicted increase in tau and increase in tau went along with cognitive change. A stepwise regression analysis in a group of 57 cognitively intact individuals between 60 and 92 years of age from the Berkeley Aging Cohort study yielded entorhinal tau PET tracer retention as the most significant predictor for subsequent cognitive decline [12], even in amyloid-negative individuals. Higher tau was related to older age and lower medial temporal grey matter volume [12]. This led to the hypothesis that entorhinal tau deposition, even in the absence of amyloidosis, may explain the episodic memory loss due to ageing [12].

### Conclusion

This study highlights the importance of baseline cognitive assessment as a predictor for cognitive change in a cognitively intact healthy older population.

## Data Availability

The datasets used and/or analysed during the current study are available from the corresponding author on reasonable request.

## Abbreviations

AAL: Automated Anatomical Labeling
AD: Alzheimer’s disease
APOE: Apolipoproteine
AVF: Animal Verbal Fluency test
AVLT: Rey’s Auditory Verbal Learning
BDNF: Brain-Derived Neurotrophic Factor
BNT: Boston Naming Test
BSRT: Buschke Selective Reminding test
CDR: Clinical Dementia Rating scale
CFI: Comparative Fit index
CL: Centiloid
DMN: Default mode network
DR: Delayed recall
F-PACK: Flemish Prevent Alzheimer’s Disease Cohort KU Leuven
FWE: Family Wise Error
FWHM: Full-width Half Maximum
HABS: Harvard Aging Brain Study
ICV: Total intracranial volume
KMO: Kaiser-Meyer-Olkin criterion
LVF: Letter Verbal Fluency test
MMSE: Mini Mental State Examination
MNI: Montreal Neurological Institute
MRI: Magnetic resonance imaging
PALPA: Psycholinguistic Assessments of Language Processing in Aphasia test
PET: Positron Emission Tomography
RMSE: Root Mean Squared Error
RMSEA: Root Mean Squared Error of Approximation
RPM: Raven’s Progressive Matrices test
SPM: Statistical Parametric Mapping
SUVR: Standardized Uptake Value Ratio
SUVR _*comp*_: Standardized Uptake Value Ratio in composite region
TI: Inversion time
TL: Total learning
TMT: Trial Making Test A and B
TR: Repetition time
